# Oleanolic Acid Acetate Alleviates Symptoms of Experimental Autoimmune Encephalomyelitis in Mice by Regulating Toll-Like Receptor 2 Signaling

**DOI:** 10.3389/fphar.2020.556391

**Published:** 2020-09-03

**Authors:** Minju Kim, Soyoung Lee, Hyungjin Lim, Jihye Lee, Ji-Young Park, Hyung-Jun Kwon, In-Chul Lee, Young-Bae Ryu, Jeongtae Kim, Taekyun Shin, Ginnae Ahn, Mun-Chual Rho, Kyungsook Jung

**Affiliations:** ^1^ Department of Marine Bio-Food Sciences, Chonnam National University, Yeosu, South Korea; ^2^ Immunoregulatory Materials Research Center, Korea Research Institute of Bioscience and Biotechnology, Jeongeup-si, South Korea; ^3^ Functional Biomaterials Research Center, Korea Research Institute of Bioscience and Biotechnology, Jeongeup-si, South Korea; ^4^ Department of Anatomy, Kosin University College of Medicine, Busan, South Korea; ^5^ Department of Veterinary Anatomy, College of Veterinary Medicine and Veterinary Medical Research Institute, Jeju National University, Jeju, South Korea

**Keywords:** multiple sclerosis, experimental autoimmune encephalomyelitis, oleanolic acid acetate, Toll-like receptor 2, inflammation

## Abstract

Toll-like receptor 2 (TLR2) is expressed by several immune cells in the central nervous system and plays an important role in neuroinflammation. TLR2 upregulation has been reported in multiple sclerosis patients and in experimental autoimmune encephalomyelitis (EAE), a mouse model for multiple sclerosis. Therefore, modulating TLR2 signaling can be an effective treatment strategy against MS. Oleanolic acid acetate (OAA) has antiinflammatory and immunomodulatory effects. Hence, this study aimed to examine the effects of OAA on TLR2 signaling and neuroinflammation in EAE. EAE was induced in C57/BL6 mice using synthesized myelin oligodendrocyte glycoprotein (MOG)_35-55_ peptide, and OAA was administered daily. Hind limb paralysis and inflammatory cell infiltration were observed in the spinal cords of EAE mice. Moreover, T-cell proliferation was significantly stimulated in splenic cells from EAE mice. The expression of proinflammatory cytokines in the spinal cord was upregulated, and their serum protein levels were increased in EAE mice. Furthermore, upregulation of TLR2 and downstream signaling molecules was observed in the spinal cord. These pathological changes were reversed by OAA treatment. Our results suggest that OAA might have promising therapeutic properties and that the TLR signaling pathway is an effective therapeutic target against multiple sclerosis.

## Introduction

Multiple sclerosis (MS) is an inflammatory, demyelinating disease of the central nervous system (CNS). Although the etiology of MS remains unknown, T helper 1 (Th1) and Th17 cells are key players in the pathogenesis of MS (Constantinescu et al., 2011). Activated Th1 and Th17 cells cross the blood-brain barrier and encounter CNS antigen-presenting cells, such as macrophages/microglia. Inflammatory cytokines secreted by these macrophages damage the myelin and axons, thus activating an inflammatory cascade in the CNS (Constantinescu et al., 2011).

Toll-like receptors (TLRs) are widely expressed by several immune cells, such as dendritic cells, T cells, B cells, monocytes, and macrophages. They play an important role in the innate immune since various stimulators, such as pathogen-associated molecular patterns (PAMPs) and damage-associated molecular patterns (DAMPs), induce lymphocyte activation (Keogh and Parker, 2011) and neuroinflammation through TLRs (Vijay, 2018; Kumar, 2019). TLR2 expression is up-regulated in oligodendrocytes, peripheral blood mononuclear cells, cerebrospinal fluid mononuclear cells, and demyelinating lesions of MS patients (Miranda-Hernandez and Baxter, 2013; Hasheminia et al., 2014; Hossain et al., 2018).

Several studies have demonstrated that microglia express TLR2 (Bsibsi et al., 2002; Sloane et al., 2010), and several TLR2 ligands, including peptidoglycans, and high mobility group box 1 (HMGB1) activate TLR2 signaling in neurons and microglia to increase the production of interleukin-1 alpha (IL-1α), IL-6, IL-12, tumor necrosis factor-alpha (TNF-α), and interferon-gamma (INF-γ) (Schrijver et al., 2001; Andersson et al., 2008). TLR2 agonists induce neuroinflammation and neuronal damage in microglia (Hoffmann et al., 2007), and TLR2 activation induced by peptidoglycan leads to proinflammatory cytokine production mediated by myeloid differentiation primary response protein 88 (MyD88) (Lin et al., 2010).

The roles of TLRs and MyD88 have been studied in experimental autoimmune encephalomyelitis (EAE), a mouse model for MS. TLR2 agonists contribute to the pathogenesis of EAE by inducing Th17 cell differentiation and promoting IL-17 production (Reynolds et al., 2010). However, in TLR2-deficient mice, EAE-induced infiltration of cluster of differentiation 4-positive (CD4^+^) T cells was observed along with decreased IL-6 production and increased number of regulatory T cells. Moreover, clinical symptoms of EAE were significantly suppressed (Miranda-Hernandez et al., 2011).

3-*O*-Acetyloleanolic acid (oleanolic acid acetate, OAA), which is a triterpenoid isolated from *Vigna angularis* (azuki bean) (Choi et al., 2013), has several pharmacological activities including antiinflammatory, antiallergic, anticancer, and suppression of osteoclast differentiation (Yoo et al., 2012; Choi et al., 2013; Kim et al., 2014; Hwang-Bo et al., 2018). Triterpenoid analogs of oleanolic acid (OA), including OAA, have been synthesized and found to be potent inhibitors of inflammation in mouse macrophages (Dinkova-Kostova et al., 2005). In addition, OA, the metabolite of OAA produced by *in vivo* hydrolysis of OAA (Kim et al., 2016), alleviated the clinical symptoms in an EAE mouse model through the inhibition of infiltration of inflammatory cells into the CNS (Martin et al., 2010; Martin et al., 2012). Furthermore, synthetic triterpenoid analogs of OA reduced Th1- and Th17-induced cytokine levels in plasma and mRNA levels in CNS tissue, peripheral lymphocytes, and mononuclear cells collected from CNS tissues in an EAE mouse model (Pareek et al., 2011). In a previous study comparing OAA and OA, OAA showed a stronger inhibitory effect against IL-6 signaling in Hep3B and U266 cell lines (Oh et al., 2014) and TLR4 signaling in THP1 blue cell line (Hwang et al., 2014) than OA. All of these findings suggest that OAA may be more effective in suppressing the pathogenesis of EAE. Since TLR2 signaling plays an important role in the pathogenesis of EAE, and the effects of OAA on TLR2 signaling have not been studied, we aimed to investigate the effects of OAA on neuroinflammation and TLR2 signaling in a mouse model of EAE.

## Materials and Methods

### Reagents

OAA was purified from *Vigna angularis* as previously described (Choi et al., 2013). Briefly, dried plant material was extracted with 95% (v/v) ethanol at 70°C. The extracts were filtered through a 0.45-mm filter and concentrated under reduced pressure to yield the ethanol extracts, which were further extracted with ethyl acetate. The ethyl acetate extract was subjected to chromatography on a silica gel column (Merck, Darmstadt, Germany) using a step gradient of an n-hexane:ethyl acetate solvent system (100:1, 80:1, 60:1, 40:1, 20:1, 10:1, and 1:1; each 1 L, v/v) to yield five fractions (H1–H5) based on thin-layer chromatography. OAA was obtained by the recrystallization of H3 in methyl alcohol, and spectroscopic analyses were performed to identify the compound.

### Mice

C57BL/6 mice were obtained from Orient Bio Inc. (Gyeonggi-do, Korea), and maintained in our animal facility. The mice were housed in ventilated cages under controlled environmental conditions (12 h light/12 h dark cycle, 21°C ± 3°C temperature, 40% ± 15% humidity). Mice were allowed free access to a standard laboratory diet (LabDiet 5053, Orient Bio Inc.) and water. All experiments were approved by the Animal Care and Use Committee of the Korea Research Institute of Bioscience and Biotechnology (KRIBB-AEC-18168).

### Induction of EAE and Administration of OAA

Seven- to eight-week-old female C57BL/6 mice were subcutaneously injected with 200 μl of myelin oligodendrocyte glycoprotein (MOG)_35-55_ peptide (NH_2_-MEVGWYRSPFSRVVHLYRN-GK-COOH; Johns Hopkins Synthesis and Sequencing Facility, Baltimore, MD, USA) (0.5 mg/ml), emulsified in complete Freund’s adjuvant (CFA) supplemented with *Mycobacterium tuberculosis* H37Ra (5 mg/ml) (Chondrex, Inc., Redmond, WA, USA) on day 0. Mice were injected intraperitoneally with 300 ng of pertussis toxin (BML-G100-0050; Enzo Life Sciences Inc., Farmingdale, NY, USA) on days 0 and 2. After immunization, the mice were weighed and observed daily for clinical signs of EAE. The progression of EAE was divided into seven clinical stages: 0, asymptomatic; 1, partial loss of tail tonicity; 2, atonic tail; 3, hind limb weakness and/or in difficulty rolling over; 4. hind limb paralysis; 5, fore limb paralysis; and 6, death due to EAE.

OAA was dissolved in 1% (w/v) carboxymethylcellulose to administer final doses of 10 mg/kg and 30 mg/kg to the mice. To determine the therapeutic effect of OAA, we administered OAA orally every day after the manifestation of the clinical symptoms from days 11 to 21 post-immunization. To evaluate the toxicity of OAA, serum biochemical analysis was performed by using DRI-CHEM NX500i (FUJIFILM Corporation, Tokyo, Japan), according to the manufacturer’s instructions. Blood samples were collected from normal or OAA-treated mice (n=8 in each group) at the end of the oral administration. Serum samples were isolated and stored at -80°C until use.

### T Cell Proliferation Assay

Proliferative responses using splenic cells were assayed as described previously (Matsumoto et al., 1996; Kim et al., 2012). Mice were sacrificed on day 21 PI (n=8). Spleens were excised immediately and weighed. Splenic mononuclear cells were dissociated and suspended in culture medium containing Dulbecco’s modified Eagle’s medium supplemented with 50 IU/ml penicillin, 50 mg/ml streptomycin, and 10% fetal bovine serum. The number of isolated mononuclear cells was counted, and 2 × 10^5^ cells/100 μl were transferred to 96-well culture plates. MOG_35-55_ and concanavalin A (Con A; Sigma Aldrich, St Louis, MO, USA) were added at final concentrations of 10 μg/ml MOG_35-55_ and 1 μg/ml Con A. After 72 h of incubation, cell proliferation was detected using the CellTiter 96^®^ Aqueous Non-Radioactive Cell Proliferation Assay (Promega Corporation, Medison, WI, USA) according to the manufacturer’s instructions. The absorbance of the reaction product was measured at 490 nm using a Varioskan LUX Multimode Microplate reader (Thermo Fisher Scientific, Waltham, MA, USA).

### Enzyme-Linked Immunosorbent Assay for Cytokine Detection

Blood was collected at the end of the experiments under isoflurane anesthesia, and serum was stored at -80°C until use. Serum IL-17 cytokine levels were measured by using a mouse IL-17 Quantikine Enzyme-Linked Immunosorbent Assay (ELISA) kit (R&D systems, Minneapolis, MN, USA). Levels of IL-6, IFN-γ, and IL-1β (BD OptEIA ELISA Set; BD Biosciences, San Diego, CA, USA) were measured following the manufacturer’s protocol and using a Varioskan LUX Microplate reader (Thermo Fisher Scientific, Waltham, MA, USA).

### Real-Time Reverse Transcription-Polymerase Chain Reaction

Total RNA (1 μg) obtained from the spinal cords was reverse transcribed into cDNA with oligo(dT)12–18 primers using PrimeScript^®^ RTase (Takara Bio Inc., Otsu, Japan), according to the manufacturer’s instructions. The reaction mixtures were amplified with TB Green™ Premix Ex Taq™ (Takara Bio Inc., Otsu, Japan) according to the manufacturer’s instructions. The following primers were used: *IL-6* forward, 5’-CAACGATGATGCACTTGCAGA-3’ and reverse, 5’-CTCCAGGTAGCTATGGTACTCCAGA-3’; *IL-1β* forward, 5’-TGCCACCTTTTGACAGTGATG-3’ and reverse, 5’-TGATGTGCTGCTGCGAGATT-3’; *TNF-α* forward, 5’-ACTCCAGGCGGTGCCTATGT-3’ and reverse, 5’-GTGAGGGTCTGGGCCATAGAA-3’; *TLR2* forward, 5’-TGTCTCCACAAGCGGGACTTC-3’ and reverse, 5’-TTGCACCACTCGCTCCGTA -3’; *MyD88* forward, 5’-TACAGGTGGCCAGAGTGGAA-3’ and reverse, 5’-GCAGTAGCAGATAAAGGCATCGAA-3’; *β-actin* forward, 5’-CATCCGTAAAGACCTCTATGCCAAC-3’, and reverse, 5’-ATGGAGCCACCGATCCACA-3’. The relative expression of the target gene was given by 2^-ΔΔCT^ and normalized to that of the endogenous reference, *β-actin.*

### Western Blot Analysis

Experimental animals were sacrificed on day 21 PI under anesthesia with isoflurane, and the spinal cords were separated and dissected. Tissues were lysed with radioimmunoprecipitation assay buffer, and the protein concentrations of the samples measured using a bicinchoninic acid (BCA) protein assay kit (Thermo Fisher Scientific, Waltham, MA, USA) according to the manufacturer’s instructions. Samples were analyzed using a 10% gel by sodium dodecyl sulfate-polyacrylamide gel electrophoresis (Bio-Rad Laboratories, Hercules, CA, USA), and the separated proteins were transferred onto nitrocellulose membranes (Millipore). After blocking nonspecific binding with 5% (w/v) bovine serum albumin in tris(hydroxymethyl)aminomethane (Tris)-buffered saline containing Tween 20 (TBS-T): 137 mM NaCl, 25 mM Tris–HCl, 2.65 mM KCl, 0.1% Tween 20, pH 7.4 at 4°C with gentle shaking overnight, we performed immunoblotting using rabbit anti-TLR2, MyD88, IL-1 receptor-associated kinase 4 (IRAK4), TNF receptor-associated factor 6 (TRAF6), phospho-IKBα (p-IKBα), and β-actin monoclonal antibodies (1:1,000 dilution, Cell Signaling Technology Inc., Denvers, MA, USA) and horseradish peroxidase-conjugated secondary antibodies (1:2,000, Cell Signaling Technology Inc., Denvers, MA, USA). Positive reactions were visualized using an enhanced Super Signal West Femto Maximum Sensitivity Substrate (Thermo Fisher Scientific, USA) and ChemiDoc imaging system (ChemiDoc XRS+ system, Bio-Rad, USA). Band intensity was quantified by ImageJ software (US National Institutes of Health, Bethesda, MD, USA).

### Hematoxylin and Eosin Staining and Immunohistochemical Staining

Spinal cord tissues were fixed in 4% (w/v) paraformaldehyde in phosphate-buffered saline (PBS, pH 7.4). The fixed tissues were embedded in paraffin and cut into 4-µm thick sections, which were stained with hematoxylin and eosin (H&E), as described previously (Ahn et al., 2012; Kim et al., 2018). Immunohistochemical analysis was performed using frozen spinal cord tissues. Briefly, the sections (5-µm thick) were mounted on slides, air-dried, fixed with cold acetone for 5 min, and blocked with 1% bovine serum albumin (BSA) in PBS for 1 h at room temperature. The slides were incubated overnight with rabbit polyclonal anti-mouse TLR2 (1:100, Thermo Fisher Scientific Inc., Waltham, MA, USA), and rat monoclonal anti-mouse CD68 (1:100, Thermo Fisher Scientific Inc., Waltham, MA, USA) antibodies at 4°C. After washing three times with PBS, the sections were labeled with Alexa 488 anti-rabbit IgG (1:200, Thermo Fisher Scientific Inc., Waltham, MA, USA) and Alexa 495 anti-rat IgG secondary antibodies (1:200, Thermo Fisher Scientific Inc., Waltham, MA, USA) for 1 h at room temperature. The nuclei were stained with DAPI in 1% BSA in PBS (4 µg/ml) and washed five times with PBS. Images were captured using a Leica fluorescence microscope (Leica DM5000B, Leica microsystems, Germany).

### Statistical Analysis

Statistical analysis was performed with GraphPad Prism Version 7 software (San Diego, CA, USA). Comparisons of two parameters were analyzed using Student’s two-tailed *t*-test. Comparisons of parameters among groups were made by one-way analysis of variance, followed by Tukey’s test. Results are expressed as mean ± standard error (SE), and p < 0.05 was considered statistically significant.

## Results

### OAA Attenuated Hind Limb Paralysis in EAE

**Figure 1:**
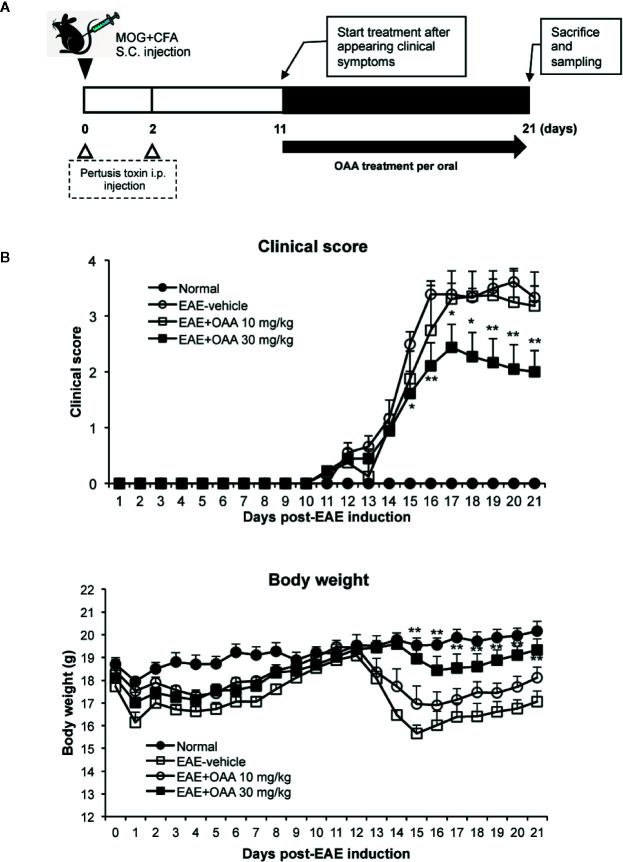
OAA attenuated clinical symptoms of EAE mice. OAA was administered after the onset of clinical symptoms **(A)**. The average scores in the OAA-treated group (black squares) were significantly lower than those of the EAE model group **(A)**. EAE pathogenesis induced weight loss was significantly recovered by OAA treatment **(B)**. Each value is shown as mean ± standard error (SE) (n=8) of the clinical score of the EAE disease, which was scored until day 21 PI. Body weight data represent means standard error (SE) (n=8). *p < 0.05, **p < 0.01 compared to vehicle-treated EAE mice. OAA, oleanolic acid acetate; EAE, experimental autoimmune encephalomyelitis; PI, postimmunization.

OAA (10 and 50 mg/kg, oral administration) has shown therapeutic effects against several immune diseases such as atopic dermatitis, arthritis (Choi et al., 2013; Oh et al., 2014), and EAE (Martin et al., 2010). Based on these results, we used oral doses of 10 and 30 mg/kg. To investigate the therapeutic effects of OAA, we administered OAA (10 and 30 mg/kg per body weight) from the onset of clinical symptoms on day 11 PI until day 21 PI (**Figure 1A**). During this period, normal control mice did not show any clinical sign such as paralysis or weight loss (**Figure 1B**). The grade of paralysis was significantly attenuated in the OAA-treated group (30 mg/kg) compared to the untreated group, whereas no therapeutic effect was observed in the 10 mg/kg treated group (**Figure 1B**). On day 21 PI, the clinical scores of OAA (30 mg/kg)-treated mice were 2 ± 0.38, and those of control mice were 3.3 ± 0.2 (**Figure 1B**). These results suggest that OAA (30 mg/kg) attenuated hind limb weakness/paralysis in the EAE mice. In addition, there was no significant difference between the control and OAA-treated groups (30 mg/kg), indicating lack of toxicity associated with oral administration of OAA (**Supplementary Table S1**). Based on these results, analysis of the therapeutic effect of OAA was conducted using the 30 mg/kg dosage.

### OAA Suppressed Antigen- and Mitogen-Specific T Cell Proliferation

**Figure 2:**
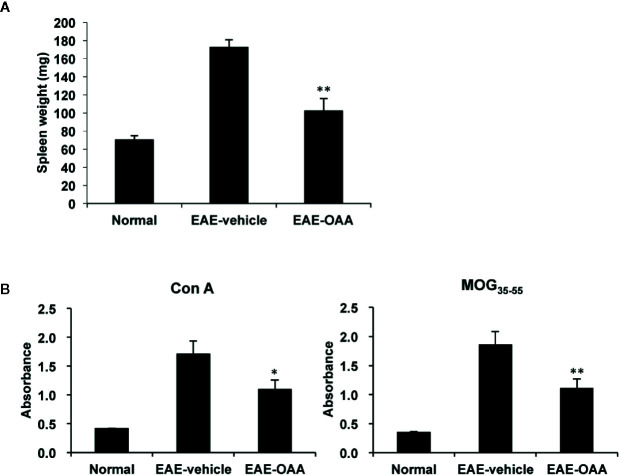
OAA inhibited proliferation of T cells and reduced spleen weight. Spleens were collected from EAE mice on day 21 PI, weighed, and cultured as single-cell suspensions. Increase in spleen weight was significantly suppressed in OAA-treated mice **(A)**. OAA treatment significantly suppressed Con A and MOG_35-55_-induced splenic T cell proliferation **(B)**. Data are represented as means ± standard error (SE) (n=8). *p < 0.05, **p < 0.01 compared to induced splenocytes from vehicle-treated EAE mice. OAA, oleanolic acid acetate; EAE, experimental autoimmune encephalomyelitis; PI, postimmunization; Con A, concanavalin A; MOG_35-55_, myelin oligodendrocyte glycoprotein peptide.

To investigate the improvement in neurological symptoms detected in OAA-treated EAE mice, we sought to examine the effects of OAA treatment (30 mg/kg) on the capacity of T cells to respond to antigens and mitogens. We collected splenocytes from OAA-treated mice and control mice and evaluated T cell response to MOG_35-55_ and Con A on day 21 PI. Immunization and the resulting lymphocyte proliferation is usually associated with an increase in the weight of the spleen. We observed a significant increase in spleen weight of EAE mice compared to those of normal mice. However, in OAA-treated mice, the spleens’ weights were significantly lower than those of control mice (n=8, p < 0.05) (**Figure 2A**). On day 21 PI, the proliferative response to MOG_35-55_ and Con A was significantly lower in OAA-treated mice than that in control mice (n=8, p < 0.01, p < 0.05) (**Figure 2B**). Furthermore, we investigated the infiltration of T cells in the spinal cords of the different mouse groups using an antibody directed against CD3, a T cell marker. CD3^+^ T cells showed more infiltration in the EAE group than in the OAA-treated EAE group; some of the CD3^+^ T cells expressed TLR2 (**Supplementary Figure S2**).

### OAA Reduced mRNA and Serum Protein Levels of Proinflammatory Cytokines

**Figure 3:**
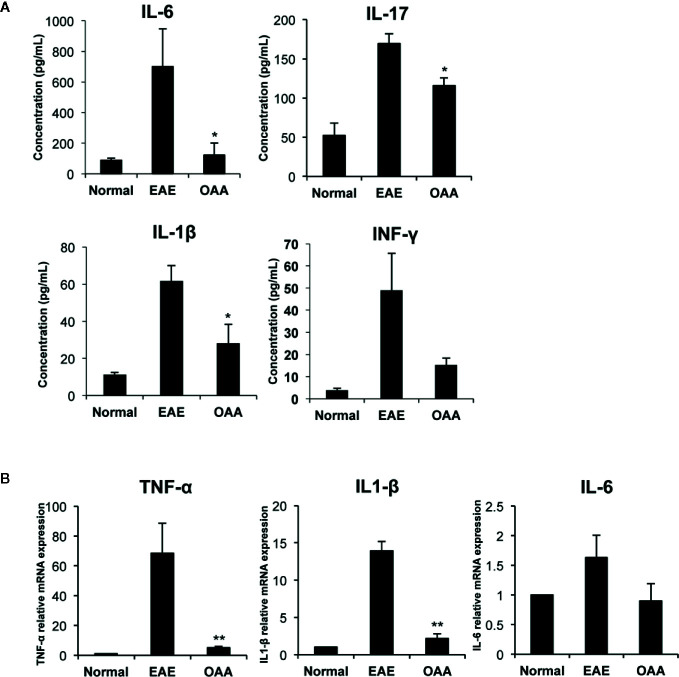
OAA regulated production of key inflammatory cytokines during EAE. Serum was collected on day 21 PI from mice, including normal, EAE mice, and OAA-treated mice with EAE. ELISA was performed to detect cytokine levels of IL-6, IL-17, IFN-γ, and IL-1β. Cytokine levels were increased in EAE mice compared to those in normal mice **(A)**. However, they were lower in the serum of OAA-treated EAE mice. mRNA levels of proinflammatory cytokines, TNF-α, IL-1β, and IL-6, were significantly suppressed in OAA-treated mice compared to those of normal control mice **(B)**. Data are represented as means ± standard error (SE) of 6 mice. *p < 0.05, **p < 0.01 compared to vehicle-treated EAE mice. OAA, oleanolic acid acetate; IL, interleukin; TNF-α, tumor necrosis factor-alpha.

To investigate the antiinflammatory effect of OAA within the CNS, we measured the mRNA levels of proinflammatory cytokines in the spinal cords of the mice. *TNF-α*, *IL-1β*, and *IL-6* mRNA was highly expressed in the CNS of the EAE mice, as compared to control mice without clinical symptoms (**Figure 3A**). The mRNA levels of the cytokines were significantly suppressed in OAA-treated mice compared to those in EAE mice (**Figure 3A**). Moreover, the serum levels of proinflammatory cytokines, such as IL-17, IL-6, and IL-1β, were highly elevated in MOG_35-55_-immunized mice and significantly suppressed by the administration of OAA (**Figure 3B**).

### Decreased Accumulation of Inflammatory Macrophages in OAA-Treated Mouse CNS

**Figure 4:**
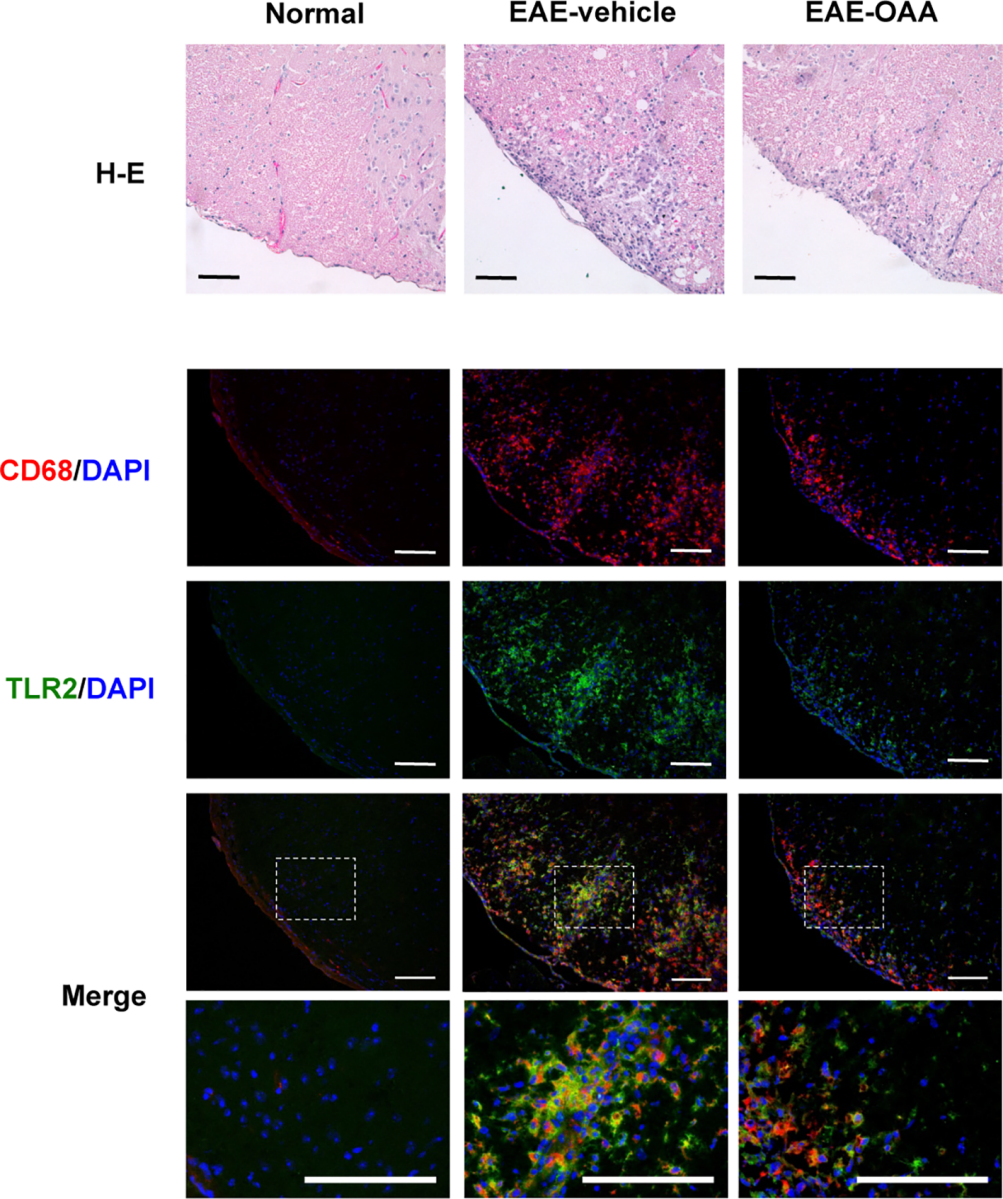
OAA suppressed infiltration of immune cells and macrophages in the spinal cords of EAE mice. H&E staining showed that OAA administration attenuated inflammatory infiltration in spinal cords compared to the infiltration observed in EAE mice (upper panel). Immunohistochemistry showed that infiltration of CD68+ macrophages was reduced in the spinal cords of OAA-treated mice (lower panel). Expression of TLR2 was elevated in EAE spinal cords and mainly expressed in CD68^+^ macrophages (lower panel). Scale bars = 100 µm. OAA, oleanolic acid acetate; EAE, experimental autoimmune encephalomyelitis; H&E, hematoxylin and eosin.

Inflammatory cells are found within the CNS in EAE mice and are associated with the clinical signs of EAE. Infiltration of inflammatory cells and the activation of macrophages in the cervical spinal cord dorsal columns have been reported in EAE (Black et al., 2006; Jones et al., 2008). Due to the behavioral improvement and the inhibition of antigen-specific proliferation by OAA, we sought to determine whether OAA might also affect inflammation in the spinal cord. Therefore, we performed histological analysis to examine inflammatory cells and macrophage infiltration within the spinal cords of OAA-treated EAE mice. To confirm the inflammatory cell infiltration, H&E staining was performed, and spinal cord tissue sections were incubated with anti-CD68, a marker for macrophages, to detect the effect of OAA on infiltrating macrophages. As shown in **Figure 4**, the accumulation of inflammatory cells and CD68^+^ macrophages was markedly suppressed in the spinal cords of OAA-treated mice. Moreover, TLR2 expression was elevated in the spinal cords of EAE model mice, which was traced to CD68^+^ macrophages that had infiltrated the spinal cord (**Figure 4**).

### Downregulation of TLR2 Signaling Pathway Molecules in OAA-Treated EAE Mice

NF-κB and AP-1 are the most common TLR-activated transcription factors involved in inflammatory responses. To determine whether TLR2 activation is modulated by OA and OAA, we investigated NF-κB and AP-1 reporter activity by using a SEAP reporter system using THP-1 Blue cells. Pam3CSK4, a well-known agonist of TLR2, induced TLR2 activation and hence, SEAP secretion in control cells, which was significantly reduced in OAA-treated cells compared to OA-treated cells at 10 µM (**Supplementary Figure S1**). Moreover, we found that OAA did not show any cytotoxicity up to 10 µM in THP-1 Blue cells (**Supplementary Figure S1**).

**Figure 5:**
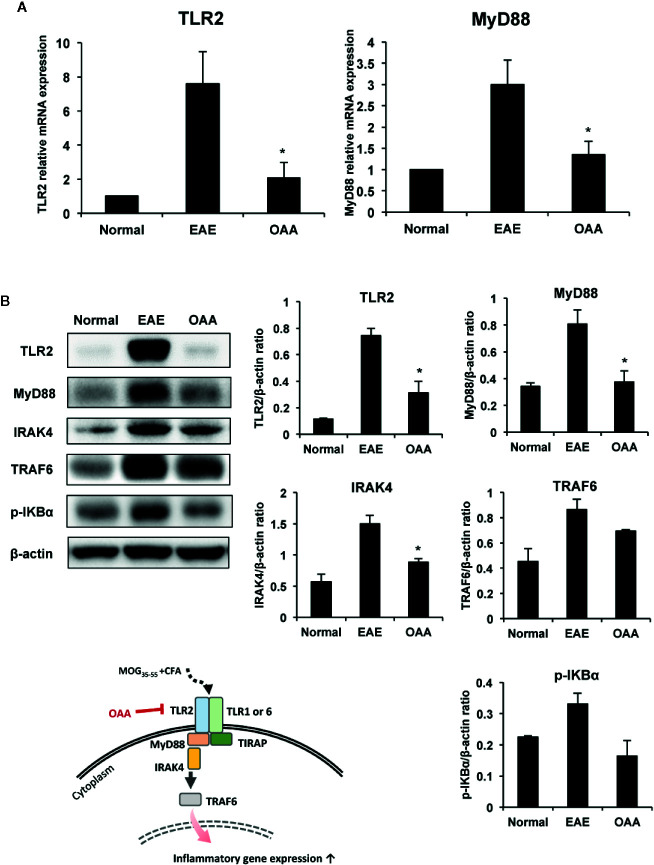
OAA downregulated the expression of TLR2 and downstream signaling molecules in the spinal cords of EAE mice. OAA downregulated TLR2 and MyD88 mRNA expression in the spinal cords **(A)**. Western blot analysis of TLR2, MyD88, IRAK4, and TRAF6 **(B)**. The expression level of TLR2, MyD88, IRAK4, and TRAF6 was significantly suppressed in OAA treated group, compared with that of EAE group **(B)**. Data are shown as mean ± standard error (SE), n=6, *p < 0.05 compared to EAE mice. OAA, oleanolic acid acetate; EAE, experimental autoimmune encephalomyelitis; TLR2, Toll-like receptor 2; MyD88, myeloid differentiation primary response protein 88; IRAK4, IL-1 receptor-associated kinase 4: TRAF6, tumor necrosis factor (TNF) receptor-associated factor 6.

As the expression of TLR2 and MyD88 plays a key role in MOG_35-55_ peptide-induced EAE pathogenesis (Miranda-Hernandez and Baxter, 2013), we examined the effects of OAA on TLR2 and MyD88 expression in the spinal cords of the immunized mice. TLR2 and MyD88 gene expression was elevated in the spinal cords of MOG_35-55_-immunized mice (**Figure 5A**).

Next, we examined the protein levels of TLR2, Myd88, IRAK4, and TRAF6 in the spinal cords of the mice using western blotting. Protein levels of TLR2 in the spinal cords were significantly higher in EAE mice than in normal control mice; however, this elevation was significantly suppressed by OAA administration (**Figure 5B**). As IRAK4 and TRAF6 are part of the TLR-induced MyD88-dependent pathway (Verstak et al., 2009; Pennini et al., 2013), we measured the protein levels of TLR2, MyD88, IRAK4, TRAF6, and p-IKBα in the spinal cords using western blot analysis. Protein levels of TLR2, MyD88, IRAK4, TRAF6, p-IKBα were higher in EAE mice than in normal mice without clinical symptoms. However, OAA-treated mice showed significantly lower protein levels of TLR2, MyD88 and IRAK4 than vehicle-treated EAE mice (**Figure 5B**). Protein levels of TRAF6 and p-IKBα were also lower in OAA-treated mice than in vehicle-treated EAE mice (**Figure 5B**). These results suggest that OAA treatment in EAE mice mitigated the inflammatory response by regulating TLR2-induced MyD88 signaling in the spinal cords (**Figure 5**).

## Discussion

OAA, a triterpenoid, has been shown to exhibit antiinflammatory activity in different models of inflammatory diseases, such as atherosclerosis, atopic dermatitis, and osteoporosis (Yoo et al., 2012; Choi et al., 2013; Kim et al., 2014; Hwang-Bo et al., 2018). Its metabolite, OA, ameliorates the clinical symptoms of EAE by inhibiting inflammatory cells in mice (Martin et al., 2010; Martin et al., 2012). As the mechanism of this amelioration and the effects of OAA on EAE remain unclear, we explored and demonstrated the suppressive effects of OAA on neuroinflammation and TLR2 signaling in MOG_35-55_-immunized EAE mice (**Figure 5**). In this study, we first identified that OAA relieves the severity of paralysis in EAE mice, through the suppression of production of proinflammatory cytokines including IL-1β, IL-6, INF-γ, and TNF-α, by regulating TLR2 signaling. By measuring SEAP secretion as an indicator of TLR2 activation in THP-1 Blue cells, we observed significant inhibition of SEAP secretion with 10 µM OAA, but not with OA (**Supplementary Figure S1A**). Based on these *in vitro* assay results, we speculated that OAA might be more effective than OA in MOG_35-55_-immunized EAE pathogenesis. Our results show that administration of OAA reduced paralysis in the MOG_35-55_ immunized EAE mice.

The precise mechanism of OAA underlying its suppression of paralysis and neuroinflammation remains to be elucidated. The TLR2-MyD88 signaling pathway plays a critical role in the pathogenesis of EAE (Verstak et al., 2009; Miranda-Hernandez et al., 2011; Zheng et al., 2019). TLR2 is expressed in CNS immune cells, such as macrophages and microglia, in MOG_35-55_-immunized EAE mice (Zekki et al., 2002; Miranda-Hernandez and Baxter, 2013) and in peripheral blood mononuclear cells in MS patients (Sloane et al., 2010). TLR2 agonists induce neuroinflammation and neuronal damage in the CNS, and TLR2 activation has been reported to cause proinflammatory cytokines production through MyD88 (Schrijver et al., 2001; Andersson et al., 2008; Racke and Drew, 2009). When TLRs recognize PAMPs or DAMPs, the MyD88 interacts with the IRAK4 and forms the MyD88-IRAK-4 complex resulting in IRAKs phosphorylation. In turns p-IRAKs interact with TRAF6, which induce the downstream signaling, such as c-Jun N-terminal kinase (JNK), mitogen-activated protein kinase (MAPK), and Phosphatidylinositol 3-Kinases (PI3K) (Xiang et al., 2015). The polarization of naïve CD4^+^T cells into Th cells is an important in the development of EAE (Hansen et al., 2006). Several studies showed that Th17 cells have a critical role in EAE, and Th17 cells secrete several proinflammatory cytokines, such as IL-17, TNF-α, that promote inflammatory responses (Becher and Segal, 2011). In addition, Th1 cells are also one of the main pathogenic T cells in EAE. MOG-specific Th1 cells induced severe EAE, and IFN-γ can be produced by Th1 cells. The TLR ligand can induce Th17 and Th1 differentiation through IL-6 mediated pathway (Shi et al., 2013). Additionally, activation of TLR-MyD88 results in a signaling cascade, which promotes the NF-κB activation (Kawai and Akira, 2007). NF-κB mediates the secretion of proinflammatory cytokines, including IL-6, and cause neuronal damage (Karin and Wildbaum, 2015; Leibowitz and Yan, 2016). Moreover, NF-κB plays crucial roles in the activation and differentiation of autoreactive Th cells *in vivo* (Hilliard et al., 1999). These results show that Th1 and Th17 cells are important immune cells to the pathogenesis of EAE, and the TLR-MyD88 signaling pathway plays a critical role in the activation of Th1 and Th17 cells.

The proliferation and infiltration of autoreactive T cells are important initiators of CNS autoimmune disease including EAE. Immunization with MOG_35-55_ leads to lymphocyte proliferation, which is associated with increased spleen weight. The number of regulatory T cells, which are related to CD4^+^ T cells, increased in the CNS of MOG-induced EAE model mice from day 7 PI to day 28 PI (Matsushita et al., 2010). An increase in the number of CD3^+^ T cells has been observed in the brain and peripheral blood of MS patients (Stinissen et al., 1995). Using the CD3 antibody, which identifies T lymphocytes and recognizes the epsilon chain of the CD3 antigen/T-cell antigen receptor (TCR) (van Dongen, et al., 1988), we observed increased infiltration of CD3^+^ T cells in the spinal cords of EAE group mice (Ouyang et al., 2014). In EAE-induced mice spinal cords, several immune cells including CD4^+^T cells, macrophages, neutrophils, and dendritic cells were increased in after day 14 post-immunization (Barthelmes et al., 2016). Furthermore, B cells increased in CNS, spleen, lymph node and peripheral blood after 21 dpi (Matsushita et al., 2010). Therefore, we estimated that the distribution of immune cells such as T cells, macrophages, neutrophils and dendritic cells in various organs including spleen, lymph node, CNS and blood are similar to the EAE pathogenesis, and these cells may be reduced by OAA administration. Administration of OAA to these immunized EAE mice significantly suppressed the proliferative response of splenic T cells to MOG_35-55_ and Con A. In addition, CD3^+^ T cell infiltration was suppressed in spinal cord tissues of OAA-treated mice (**Supplementary Figure S2**). These observations suggest that OAA plays a critical role in the inhibition of the development of pathogenesis by regulating lymphocyte proliferation and infiltration.

Levels of proinflammatory cytokines, including IL-6, IL-17, IFN-γ, and IL-1β, have been reported to be higher in the CNS and sera of EAE mice (Borjini et al., 2016; Palle et al., 2017). In this study, we found that administration of OAA suppressed the up-regulation of proinflammatory cytokines in the sera of the treated mice compared to those of the MOG_35-55_-immunized EAE mice. Levels of mRNA of proinflammatory cytokines, such as *TNF-α*, *IL-1β*, and *IL-6*, were also significantly inhibited in the spinal cords of EAE mice following OAA treatment. After OAA administration in MOG_35-55_-immunized EAE mice, infiltration of inflammatory cells and CD68^+^ macrophages were significantly reduced, demonstrating the alleviation of EAE clinically and histologically. Thus, these observations indicate that OAA mitigated neuroinflammation through the suppression of activation of macrophages.

In EAE spinal cord, TLR2 signaling may be activated upon stimulation by MOG_35-55_. In mice with TLR2 and MyD88 deficiency, the suppression of CD4^+^ T cell infiltration in the CNS was found to mitigate susceptibility to MOG_35-55_-induced EAE and the absence of TLR2 (Miranda-Hernandez et al., 2011). Furthermore, increased availability of TLR2 in epithelial cells corresponded to an increase in proinflammatory responses seen in the airways of cystic fibrosis patients (Muir et al., 2004). However, TLR2 has both pathogenic and protective effects in MS patients (Deerhake et al., 2019). We had previously demonstrated that OAA decreased the levels of proinflammatory cytokines and chemokines, including IL-1β and IL-8, in THP-1 Blue cells (Lim et al., 2019). Therefore, we postulated that OAA treatment in EAE-induced mice alleviated the clinical signs and inflammatory responses *via* the TLR2 signaling pathway.

As our hypothesis (**Figure 5**), TLR2 activation contributes to neuroinflammation and EAE pathogenesis, while our results show the reduced levels of TLR2 related molecules in the spinal cords of OAA-treated EAE mice. MOG_35-55_ immunization induces TLR2 activation in infiltrated macrophages, which leads to increased production of proinflammatory cytokines—key events in EAE pathogenesis. OAA suppresses this inflammatory immune response in the CNS by inhibiting TLR2 signaling and hence, could be a useful therapeutic agent for ameliorating the clinical symptoms of EAE and ultimately, MS.

## Data Availability Statement

The raw data supporting the conclusions of this article will be made available by the authors, without undue reservation.

## Ethics Statement

The animal study was reviewed and approved by Animal Care and Use Committee of the Korea Research Institute of Bioscience and Biotechnology (KRIBB-AEC-18168).

## Author Contributions

Conceptualization: M-CR and KJ. Experimental design: MK, SL, and KJ. Data curation; MK, GA, SL, and KJ. Investigation: MK, SL, HL, JL, and KJ. Funding acquisition: M-CR, SL, J-YP, H-JK, Y-BR, I-CL, and KJ. Methodology: MK, SL, TS, JK, and KJ. Project administration: MK, SL, and KJ. Supervision: M-CR and KJ. Writing—original draft: MK, SL, and KJ. Writing—review and editing: TS, JK, and KJ.

## Funding

This research was supported by a grant from the KRIBB Research Initiative Program (KGM5242012).

## Conflict of Interest

The authors declare that the research was conducted in the absence of any commercial or financial relationships that could be construed as a potential conflict of interest.
