# Supplementary material for: Oleanolic Acid Acetate Alleviates Symptoms of Experimental Autoimmune Encephalomyelitis in Mice by Regulating Toll-Like Receptor 2 Signaling
Source: Front Pharmacol. 2020 Sep 3;11:556391. doi: 10.3389/fphar.2020.556391 (PMC7494849; doi:10.3389/fphar.2020.556391)
Supplement: Supplementary file 1 [file DataSheet_1.docx]

Supplementary Material

# Supplementary Data

**Reagents**

OA (purity > 97%) was purchased from Sigma-Aldrich (cat. No. O5504, St Louis, MO, USA). OAA was purified from *Vigna angularis* as previously described (Choi et al., 2013). Briefly, dried plant material was extracted with 95% (v/v) ethanol at 70°C. The extracts were filtered through a 0.45-mm filter and concentrated under reduced pressure to yield the ethanol extracts, which were further extracted with ethyl acetate. The ethyl acetate extract was subjected to chromatography on a silica gel column (Merck, Darmstadt, Germany) using a step gradient of an n-hexane:ethyl acetate solvent system (100:1, 80:1, 60:1, 40:1, 20:1, 10:1, and 1:1; each 1 L, v/v) to yield 5 fractions (H1–H5) based on thin-layer chromatography. OAA was obtained by the recrystallization of H3 in methyl alcohol, and spectroscopic analyses were performed to identify the compound. For use in cell culture experiments, OAA was dissolved in dimethyl sulfoxide to obtain a solution of 10 mM concentration.

***In Vitro* Assay for TLR2 Inhibition**

THP1-Blue™ cells, stably expressing a secreted embryonic alkaline phosphatase (SEAP) reporter inducible by nuclear factor-kappa B and activator protein-1, were purchased from InvivoGen (InvivoGen, San Diego, CA, USA). TLR2 activation was measured in terms of the SEAP secretion. Briefly, THP1-Blue cells were seeded in flat-bottom 96-well plates at a density of 2 × 10^5^ cells/well, and treated with OAA (1, 3, 10 µM) for 1 h. The synthetic lipopeptide Pam3CSK4 (50 ng/mL, InvivoGen, San Diego, CA, USA), a TLR2-specific agonist, was incubated with the cells for 18 h at 37°C in a humidified atmosphere containing 5% CO_2_. Cell supernatants (20 µL) were transferred to new 96-well plates and mixed with 180 µL/well of QUANTI-Blue Solution (InvivoGen, San Diego, CA, USA), an SEAP detection reagent. After incubation at 37°C for 1 h, SEAP levels were determined using a spectrophotometer at 650 nm. Cell viability was determined using water-soluble tetrazolium salt (WST)-based cell cytotoxicity assay kit (EZ-CYTOX, Daeil Lab Service, Seoul, Korea) according to the manufacturer’s instructions.

## Immunohistochemical Staining

Immunohistochemical analysis was performed using frozen spinal cord tissues. Briefly, the sections (5 µm thick) were air-dried and fixed with cold acetone for 5 minutes and were then blocked with the 1% BSA in PBS for 1 h at room temperature. The slides were incubated with rabbit polyclonal anti-mouse TLR2 (1:100, Thermo Fisher Scientific Inc., Waltham, MA, USA), and hamster monoclonal anti-mouse CD3 (1:100, BD Pharmingen, MA, USA) antibodies at 4°C overnight. After washing 3 times with PBS, the sections were labeled with Alexa 488 anti-hamster IgG (1:200, Thermo Fisher Scientific Inc., Waltham, MA, USA) and Alexa 495 anti-rabbit IgG secondary antibodies (1:200, Thermo Fisher Scientific Inc., Waltham, MA, USA) for 1 h at room temperature. Nuclei were stained with DAPI in 1% BSA in PBS (4 µg/mL concentration) and washed with PBS, five times. Images were taken on Leica fluorescence microscope (Leica DM5000B, Leica microsystems, Germany).

**Serum biochemistry**

Serum markers were analyzed using a dry chemistry analyzer (FUJI DRI-CHEM NX500i, Fujifilm, Tokyo, Japan) according to the manufacturer’s instructions. Blood samples were collected from control and OAA-treated mice (n = 8 in each group) at the end of the experiment. Serum samples were stored at -80°C until use.

# Supplementary Figures and Tables

## Supplementary Figures


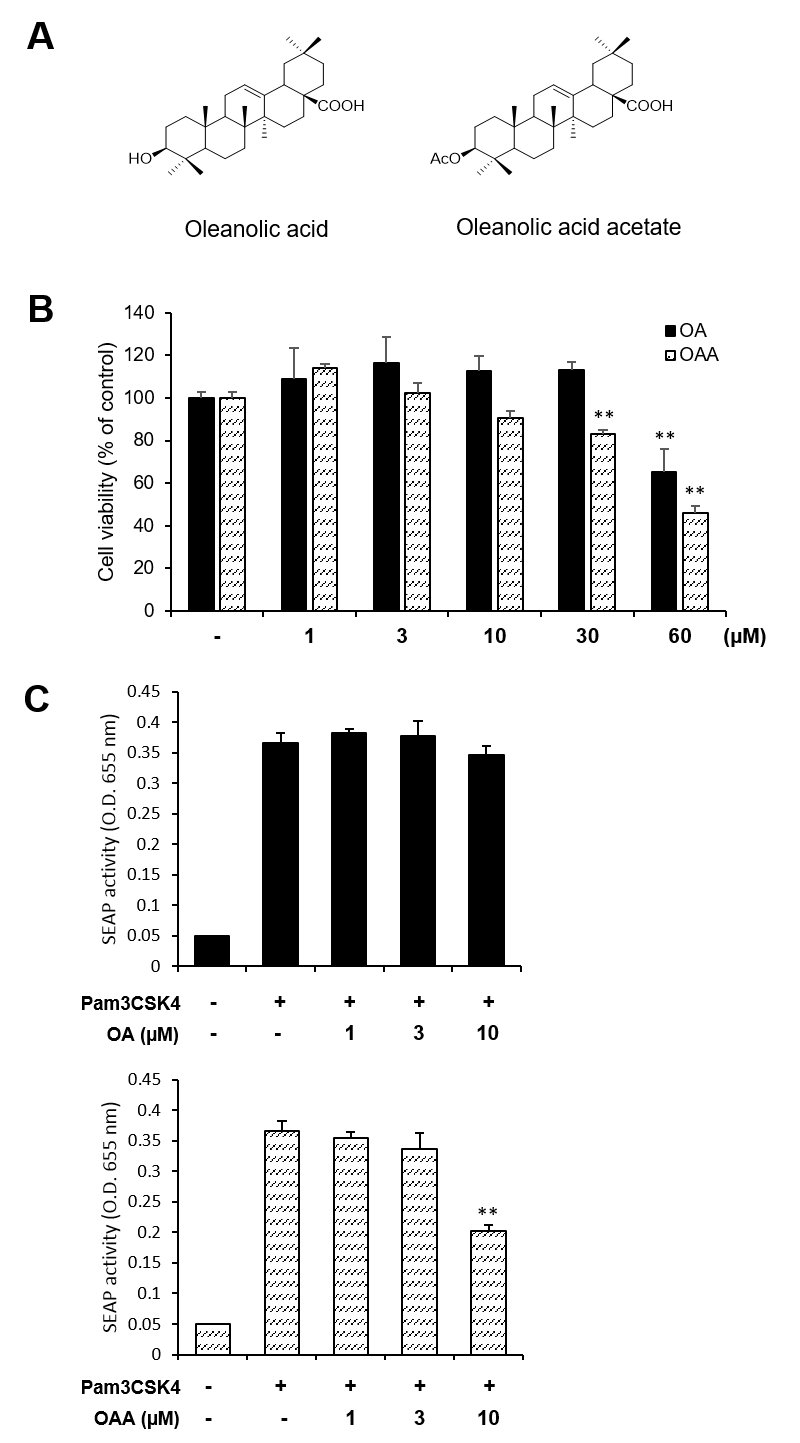


**Supplementary** **Figure S1. Inhibition of TLR2 activation by OA and OAA in THP-1 Blue cells.** Chemical structure of OA and OAA (A). Viability was measured in OA- and OAA-treated cells after 24 h treatment using WST-1 assay (B). Cells were incubated with OA (0, 1, 3, 10 µM) or OAA (0, 1, 3, 10 µM) for 1 h before stimulation with Pam3CSK4 (50 ng/mL) for 18 h, and the secretion of SEAP was measured using QUANTI-Blue (C). Data are represented as means ± standard error (SE) of 3 different experiments. **p < 0.01 compared to medium alone. OA, oleanolic acid; OAA, oleanolic acid acetate; SEAP, secreted embryonic alkaline phosphatase.


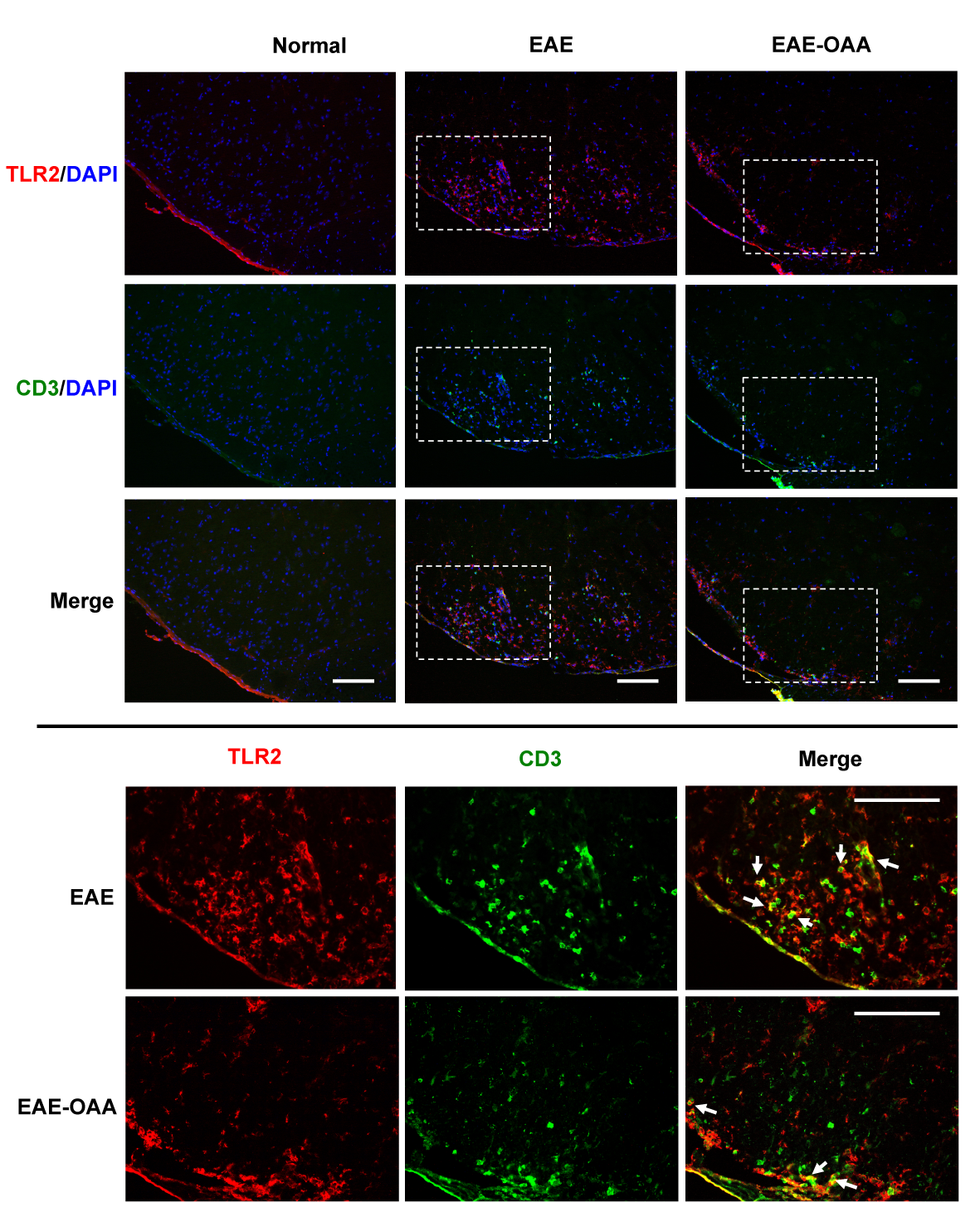


**Supplementary Figure S2. OAA suppressed infiltration of T cells in the spinal cords of EAE mice.** Immunohistochemistry showed that infiltration of CD3^+^ T cells was reduced in the spinal cords of OAA-treated mice (upper panel). Expression of TLR2 were elevated in EAE spinal cords, and TLR2 expressed in some CD3^+^ T cells (lower panel, arrows). Scale bars = 100 µm.

## Supplementary Table

**Supplementary TABLE S1. Serum biochemistry revealed lack of toxicity of OAA.**

|  | Normal mice | OAA-treated mice |
| --- | --- | --- |
| ALB (g/dL) | 1.9 ± 0.23 | 1.5 ± 0.1 |
| ALT (U/L) | 18.7 ± 1.53 | 13.0 ± 9.1 |
| AST (U/L) | 81.7 ± 22 | 102.3 ± 8.5 |
| BUN (mg/dL) | 18.1 ± 1.75 | 24.5 ± 8.58 |
| CRE (mg/dL) | 0.1 ± 0 | 0.1 ± 0 |
| TP (g/dL) | 3.7 ± 0.1 | 3.5 ± 0.12 |
| TBIL (mg/dL) | 0.4 ± 0.06 | 0.4 ± 0 |

No significant differences were observed between the groups, indicating lack of toxicity of OAA. Data represent means ± standard error (SE) of 8 mice. OAA, oleanolic acid acetate; ALB, albumin; ALT, alanine aminotransferase; AST, aspartate aminotransferase; BUN. Blood urea nitrogen; CRE, creatinine; TP, total protein; TBIL, total bilirubin.
